# Development and evaluation of an evidence-based medicine module in the undergraduate medical curriculum

**DOI:** 10.1186/s12909-020-02181-7

**Published:** 2020-08-06

**Authors:** Abeer Salem Al Shahrani

**Affiliations:** Department of Clinical Sciences, College of Medicine, Princess Nourah Bint Abdulrahman University, Riyadh, Saudi Arabia

**Keywords:** Evidence-based medicine, EBM, Medical students, Undergraduates, Curriculum, Course

## Abstract

**Background:**

Evidence-based medicine (EBM) is a core competence in both undergraduate and postgraduate medical curricula. However, its integration into curricula varies widely. Our study will help medical colleges develop, implement and evaluate their EBM courses. We assessed the effectiveness of workshops in improving critical appraisal skills among medical students.

**Methods:**

A before-and-after study design without a control group was used. A 5-week short EBM module including lectures, workshops, and online search sessions was conducted with 52 fourth-year medical students during their primary healthcare course at the College of Medicine, Princess Nourah bint Abdulrahman University. Statistical analysis was performed using SPSS statistical software (version 20, SPSS Inc., Chicago, US). Parametric tests as well as Student’s paired t-test for pre- and post-test comparisons were used.

**Results:**

Forty-nine (49) participants completed the pre- and post-training Fresno tests, and 44.9% of the participants had a GPA of 4.0 or higher. The mean Fresno test score increased from 45.63 (SD 21.89) on the pre-test to 64.49 (SD 33.31) on the post-test, with significant improvements in the following items: search strategies, relevance, internal validity, magnitude and significance of results, statistical values of diagnosis studies (sensitivity, specificity, and LR), statistical values of therapy studies (ARR, RRR, and NNT), and best study design for diagnosis and prognosis (*P* < 0.05).

**Conclusion:**

This study supports that a short course in EBM that is incorporated into the undergraduate curriculum, especially in the clinical years, might be effective in improving medical students’ knowledge and skills in EBM. However, prospective studies are necessary to assess the long-term impact of these interventions and ultimately their effectiveness for clinical decision making.

## Background

Evidence-based medicine (EBM) was first introduced in early 1990 and was defined by Sacket et al. [1] as “the conscientious, explicit, and judicious use of current best evidence in making decisions about the care of individual patients.” The principles of EBM include asking the appropriate clinical question, searching related and relevant clinical data, appraising the data, applying the data to the appropriate clinical scenario, and finally evaluating the data [2]. This concept has been adopted by many allied healthcare professionals, and the Sicily statement of evidence-based practice proposed the change to evidence-based practice (EBP) instead of EBM [3]. Thus, EBM is considered one of the important revolutions in healthcare education and practice, and it has become a core competence in both undergraduate and postgraduate medical curricula [4, 5]. However, there are no clear recommendations on when to initiate teaching on EBM, as indicated by a series of studies that showed no difference in EBP competence between undergraduates and postgraduate medical students [6].

Thus, the integration of EBM into the undergraduate curriculum varies from medical school to another [7]. In addition, there is debate about the most effective training method for EBM for undergraduate students. Different types of teaching methods, such as flipped classrooms, journal clubs, seminars and morning reports, have been reported in the literature [8–10]. Furthermore, some studies have proposed that EBM may be offered online or in a blended learning format [11, 12]. Moreover, a number of studies have recommended the shift to more student-centred and problem-based learning [13]. These educational activities have shown an impact on EBP knowledge, attitudes and skills and, eventually, might affect patient outcomes and the quality of health care [14]. However, the degree of impact on healthcare practice and sustainability due to the changes after these activities have been implemented is unclear [15].

Assessment and evaluation of EBP competence is a complex task to achieve through the use of a single method [16]. A few validated tools exist to assess competence, and they have been widely used. Among the most common tools are the Berlin and Fresno tests [17, 18], but similar validated tools to determine the extent to which attitudes change after an educational intervention are lacking. Moreover, studies that rely on student self-reports as measurement tools have shown that self-reports are not a reliable method for measuring long-term changes in the attitudes or behaviour of medical students [19, 20].

This study needs to be published because there are few published studies on EBM teaching in developing countries [21]. In addition, one local study on medical students from different colleges in the Kingdom of Saudi Arabia reported low levels of knowledge and attitudes towards EBM [22], which calls for a well-structured incorporation of EBM as a major competence into the undergraduate curriculum. Moreover, it has been suggested that the emphasis should not be on teaching the science of EBM but rather on its practical application to patient care and that the curriculum would be better named the Knowledge Translation Curriculum [23].

The College of Medicine at Princess Nourah bint Abdulrahman University (PNU) is a newly established medical college in the Kingdom of Saudi Arabia. To fulfil the college mission, efforts have been made to improve the curriculum in general and the EBM module in particular since EBM is one of the core programme competencies. Thus, for the past 4 years, multiple changes in the module content and assessment methods have taken place. This article describes an initiative to develop, implement and evaluate a short EBM module for Year 4 students of the MBBS programme. In addition, the article assesses the effectiveness of a hands-on workshop on EBM using a validated tool (the Fresno test) and assesses students’ overall performance using an authentic assignment.

## Methods

### Context

Our curriculum is hybrid and problem-based, and it takes students over 5 years to complete.

The fourth year consists of eight clinical courses that range between 5 and 9 weeks in duration, including primary health care, ENT/OPHTHA, OB/GYN, orthopaedics, dermatology, psychiatry and anaesthesia courses.

The EBM module was first introduced as part of the primary health care course among the first cohort in 2015/16, and it has continued to be part of the 4th year curriculum since then. The primary health care course was the first clinical rotation in the fourth year. It introduced the students to not only EBM but also communication and consultation skills, which are important foundations for the medical profession. The EBM module was included in the fourth year, which is taken after the medical research/epidemiology course that is offered in the third year. The placement of the module was deliberate to ensure that students would have a foundation in study design, research methodology and basic biostatistics prior to taking the EBM module. The objectives of the module included the following: to develop relevant knowledge and skills in framing questions in the PICO format, conducting database searches and critically appraising findings and be able to demonstrate these steps of EBM. That is, by the end of the module, the students were expected to be able formulate a PICO question based on a clinical scenario, search online for the relevant studies, critically appraise the findings for their validity and appropriateness and discuss their applicability. These skills were assessed with the use of a graded rubric for both written (report) and verbal (presentation) assignments. The module was taught by faculty members who were specialized in family medicine (2), community medicine (1) and public health. (1) All faculty were trained in EBM teaching either in the Centre for Evidence-Based Medicine at the University of Oxford or at McMaster University and had been trainers at the National & Gulf Centre for Evidence-Based Health Practice in Riyadh, Saudi Arabia.

The content of the module is shown in Table 1. The module included the following elements. In week 1, 50- to 100-min lectures were delivered on one to three mornings throughout the week. Additionally, 50–100-min interactive sessions were used for online database searching. During week 2, 75–150-min small-group workshops were conducted on critical appraisal skills using therapy and diagnosis papers that were chosen by the trainers. In weeks 3 and 4, students were assigned to small groups supervised by faculty to work on their own assignments. In week 5, each student’s skills in presenting an EBM topic were assessed; each student submitted a detailed report that was standardized and formatted to cover the 5 A's EBM steps (Ask, Acquire, Appraise, Apply and Assess). Students were asked to submit the full-text article used in the assignment as well as the critical appraisal sheet.

**Table 1:** Content of the EBM module

Topic	Learning Objectives	Teaching Method/Duration	Timing
EBM I: Introduction	• Define EBM • List the EBM steps • Formulate answerable clinical questions (PICO)	Lecture/100 min	Once/Week 1
EBM II: Literature search	• Translate PICO questions into a search strategy • Demonstrate the EBM resources search	Interactive tutorial/50–100 min	Once/Week 1
Critical Appraisal Skills I	• Understand the concepts of critical appraisal (diagnosis and therapy)	Lecture/50 min	3 times/Week 1
Critical Appraisal Skills II		Lecture/50 min	
Critical Appraisal Skills III		Lecture/50 min	
EBM Workshop I	• Critically appraise an article on therapy (RCT–SR&MA) using a McMaster worksheet	Hands-on workshop/150 min	Once/Week 2
EBM Workshop II	• Critically appraise an article on diagnosis using a McMaster worksheet	Hands-on workshop/75 min	Once/Week 2
EBM Assignment	• Demonstrate EBM steps for clinical questions selected by the student under faculty supervision	EBM Presentation and report	SDL^a^/Week 3–4

^a^*SDL* self-directed learning

In this study, we did not test the validity, inter-rater agreement, internal consistency, acceptability and feasibility due to the small sample size. However, we collected the relevant data for consideration for future publication about adapting authentic assignment as a method of EBM skills assessment. Moreover, since we started teaching the EBM module, it has evolved with time and has undergone modifications, whether in teaching strategy or assessment method. Recently, we added two small group workshops wherein students choose their own clinical question until they retrieve articles they have chosen for their assignment and appraise them; then, the students are distributed into pairs to enhance peer learning. Currently, with the Covid-19 pandemic, we have been conducting a virtual module that is different and interesting.

### Study design

No control group was included in this before-and-after study, as the participants were the first cohort who received the educational intervention. This was our first experience teaching EBM in our curriculum. Moreover, the EBM module has evolved over time, making it difficult to compare between cohorts.

### Sample size

All fourth-year medical students were included (*n* = 52).

### Sampling technique

Not applicable.

### Data collection methods, instruments used, measurements

A before-and-after study design was used. A 5-week short EBM module including lectures, workshops, and online search sessions was conducted with fifty-two fourth-year medical students during their primary healthcare course at the College of Medicine, PNU, from the first of September until mid-December 2016. We used the Fresno test to assess the effectiveness of a hands-on workshop on EBM knowledge and skills gain. Overall, students’ performance was assessed by EBM assignment at the end of the module. The pre-test was administered in the second week of the module after ending the theoretical part and immediately before the critical appraisal workshops. This timing was chosen because EBM is a relatively new subject for students, and on the other hand, we would like to assess whether workshops will improve their skills gain or not. The post-test was administered during week 3 after the teaching of the module, including the workshops, was completed. Each student had a code number that replaced her name on the pre- and post-test papers to ensure that the students’ identities were protected. Moreover, to avoid inter-rater bias, an experienced faculty member in EBM was responsible for grading both the pre- and post-test papers. However, none of the scores (pre/post) were counted as part of the students’ assessment or final grade. In weeks 3 to 5, students were asked to work on their EBM assignment under faculty supervision. Therefore, each student was expected to come up with a clinical question/scenario from either their observation or interest and then work on EBM steps as they have been taught during the workshops. They may also visit or contact their supervisors if they face difficulties on this task. By the end of the course, i.e., week 5, all students should submit their work in report format and present their topic in front of the group. One faculty member was responsible for marking the EBM reports using a standardized format. Two other faculty members were invited to assess students’ EBM presentation skills using a standardized rubric. Their performance in the assignment accounted for 10% of their primary health care course grade.

### Statistical analysis

Statistical analysis was performed using SPSS statistical software (version 20, SPSS Inc., Chicago, US). The effectiveness of the EBM workshop was assessed based on the differences in the total and subtotal pre- and post-training Fresno test scores, which were the primary outcome. Parametric tests were used to test the study hypotheses, such as the mean, standard deviation, and confidence interval (CI %). Comparison of the total Fresno pre- and post-test scores was conducted using Student’s paired t-test to determine whether there was a statistically significant difference in the students’ performance before and after the intervention. A *P* value .05 was considered significant.

### Reliability of the Fresno test

The Fresno test is an objective, comprehensive tool that consists of 12 items that cover basic knowledge and skills in EBM [18]. The test includes two clinical scenarios with open-ended questions. Participants are required to complete the four key steps of the EBP process to adequately answer the open-ended questions related to the clinical scenarios. In addition, two questions are related to statistics for observational and experimental studies. We omitted some items in the calculation section because 98% of students left them blank on the pre-test. Therefore, the total Fresno score in this study ranged from 0 to 204.

Cronbach’s alpha and the item-total correlation were used to determine internal reliability. Cronbach’s alpha was used as the index of internal consistency of the test, with an acceptable range from .7 to .95. The item-total correlation was used to determine the reliability of each scale, with an acceptable value of 0.2 or higher. The lowest item-total correlation was .263, which is above the acceptable value (item 9). As shown in Table 2, Cronbach’s alpha was .775, which is statistically acceptable.

**Table 2:** Reliability results and item-total statistics

	Corrected Item-Total Correlation	Cronbach’s Alpha If Item Deleted
Item 1A	.415	.763
Item 1B	.401	.763
Item 2	.455	.760
Item 3	.443	.757
Item 4	.523	.747
Item 5	.483	.754
Item 6	.497	.755
Item 7	.556	.745
Item 8	.500	.752
Item 9	.263	.775
Item 10	.268	.774
Item 11	.369	.768
Item 12	.351	.768
**Total items**		**.775**

## Results

**Fig. 1:**
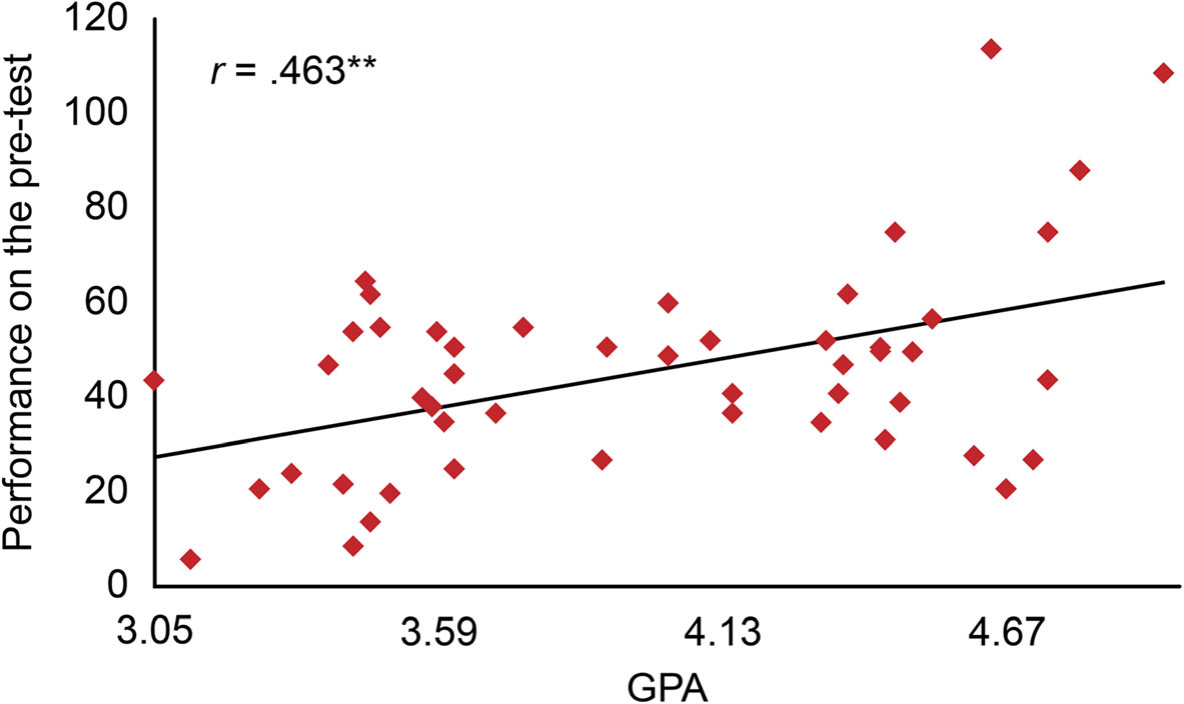
Students’ performance on the pre-test (*n* = 49)

**Fig. 2:**
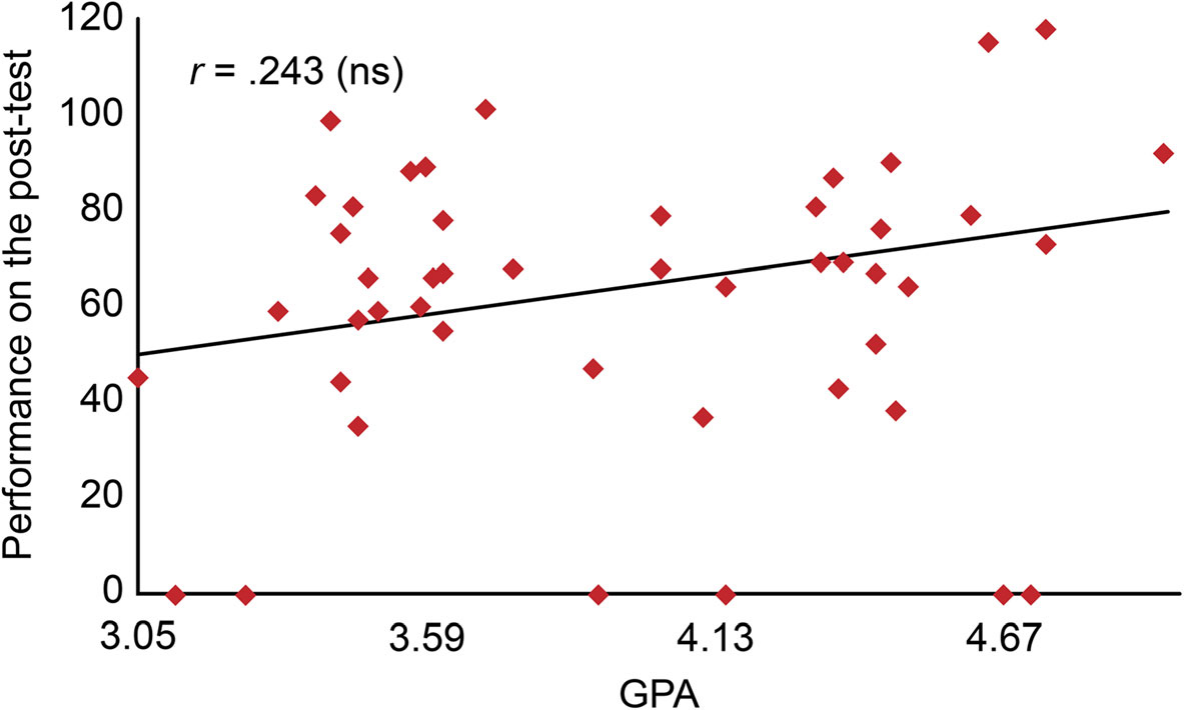
Students’ performance on the post-test (*n* = 49)

After data cleaning, forty-nine (49) participants who completed the pre- and post-Fresno tests were included in the analysis. Fifteen students (30.6%) had a GPA ranging between 3.05 and 3.58 out of 5, while twelve (24.5%) had a GPA in the range of 3.59 to 4.12, and twenty-two (44.9%) had a GPA of 4.13 or higher. Overall, students’ performance improved on the post-test; the improvement was statistically significant even for those with a low GPA, as shown in Figs. 1 and 2.

The students’ scores were classified into 4 categories: poor (0–50), average (51–101), good (102–152) and excellent (153–204). Regarding student performance on the pre-test, the majority of students had a poor score of 67.3, 28.5% had an average score, and only 4% had a good score. There was a statistically significant difference between the pre- and post-tests; on the post-test, the majority had an average score (63.3%), followed by 26.5% who had a poor score, 8% who had a good score and 2% who had an excellent score (chi-square = 16.406, DF = 2, *P* value = 0.000).

A comparison using a paired t-test shows that the mean Fresno test score increased from 45.63 (SD 21.89) on the pre-test to 64.49 (SD 33.31) on the post-test, with statistically significant improvements seen in the following items: Searching strategies, Relevance, Internal validity, Magnitude & significance of results, Statistical values of diagnosis studies (sensitivity, specificity, and LR), Statistical values of therapy studies (ARR, RRR, and NNT), Best study design for diagnosis and Best study design for prognosis (*P* < 0.05) (as shown in Table 3). These results indicate the effectiveness of hands-on workshops in improving technical skills related to EBM, such as calculations and search strategies.

**Table 3:** Fresno test score (*n* = 49) based on the results of the paired samples t-test

Item #	Area of knowledge	Test	Mean (95% CI)	SD	T value	*P* value
1 A & B	Formulation of clinical questions (PICO format)	Pre-test	7.28 (6.58–7.980)	2.44	1.196	.24^(ns)^
		Post-test	7.84 (6.84–8.84)	3.49		
2	Sources of evidence	Pre-test	3.51 (*2.84*–*4.18*)	2.33	1.741	.08^(ns)^
		Post-test	4.16 (*3.34*–*4.99*)	2.88		
3	Searching strategies	Pre-test	4.57 (*3.33*–*5.81*)	4.32	2.355	.02*
		Post-test	6.37 (*4.66*–*8.08*)	5.95		
4	Study design	Pre-test	10.65 (*9.19*–*12.11*)	5.09	1.271	.21^(ns)^
		Post-test	11.57 (*9.94*–*13.20*)	5.68		
5	Relevance	Pre-test	1.63 *(.84–2.43*)	2.77	3.577	.001**
		Post-test	3.57 (*2.47*–*4.67*)	3.82		
6	Internal validity	Pre-test	1.98 (.58–3.38)	4.86	2.291	.027*
		Post-test	4.16 (1.81–6.52)	7.64		
7	Magnitude & significance of results	Pre-test	2.94 (1.16–4.72)	6.21	3.326	.002**
		Post-test	5.94 (4.17–7.71)	6.17		
8	Statistical values of diagnosis studies (sensitivity, specificity, and LR)	Pre-test	.49 (−.11–1.09)	2.10	7.247	.000**
		Post-test	4.41 (3.38–5.44)	3.58		
9	Statistical values of therapy studies (ARR, RRR, and NNT)	Pre-test	1.47 (.33–.61)	3.97	2.424	.019*
		Post-test	3.35 (2.08–4.62)	4.42		
10	Confidence interval estimation	Pre-test	.33 (.01–.64)	1.11	1.950	.058^(ns)^
		Post-test	.74 (.26–1.23)	1.58		
11	Best study design for diagnosis	Pre-test	1.96 (1.38–2.54)	2.02	3.947	.000**
		Post-test	3.34 (2.89–3.81)	1.49		
12	Best study design for prognosis	Pre-test	1.55 (.99–2.12)	1.97	2.549	.01*
		Post-test	2.51 (1.91–3.11)	1.96		

NB: * Significant at 0.05, ** Significant at 0.01, *ns* Not significant

**Fig. 3:**
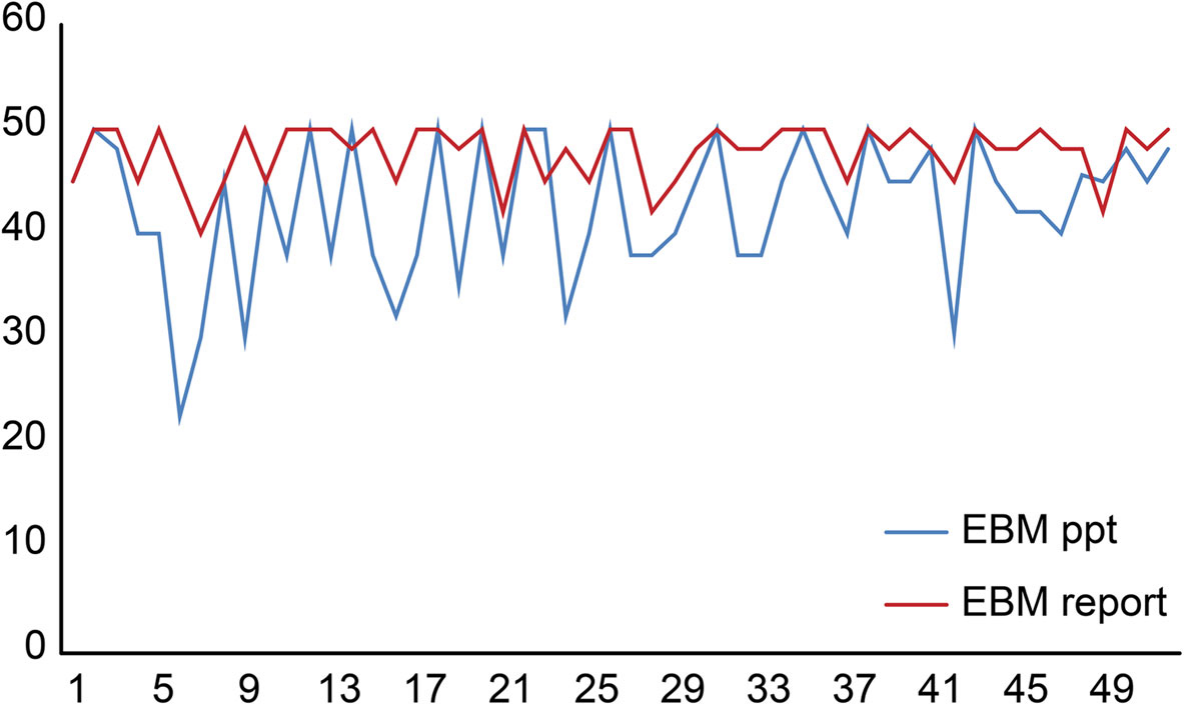
Students’ performance on the EBM assignments (presentation and report) (*n* = 52)

Our secondary outcome was students’ overall performance on the EBM assignment. This outcome was assessed with two methods, an oral presentation graded from 0 to 50% and an EBM report graded from 0 to 50%, as shown in Fig. 3. Overall, students performed well on both assignments, with mean scores of 42.5 and 47.7 for the presentation and report, respectively.

## Discussion

The Fresno test is a reliable and valid tool for the assessment of knowledge and skills on the three key EBP steps: asking focused questions, searching for relevant resources, and critically appraising validity and appropriateness. The test was a good tool to assess changes due to the educational intervention, i.e., an EBM workshop, in this study. However, the item difficulty was high to very high. Despite an overall improvement on the post-test, few students were able to obtain good total scores. This might be explained by the following: EBM is relatively a new subject for the students, and they had just finished the introductory lectures. Additionally, students are usually exam oriented, so since this test was not part of their course assessment, the results would not affect their final grade. Therefore, they were relaxed and likely not prepared as if it was a real exam. Moreover, we expect that total scores would have been lower if we had retained the items related to advanced calculations for diagnosis and therapy, which were given little focus during theoretical teaching. However, few students have tried to answer calculation questions by writing down the equations only. However, this was not counted as complete answer as per the FRESNO test grading rubric. Therefore, we recommend customizing the FRESNO test according to what has been taught in the module to obtain results that reflect the actual knowledge and skills gained. Our study findings suggest that it would be better to conduct the test with more experienced groups, such as medical residents, similar to the original cohort [18]. Notably, answering open-ended questions is challenging for undergraduate students, especially if the questions have multiple parts, such as items 2 and 3. Therefore, in our case, the majority of students answered only the first part of the questions. However, we are not sure if the students unintentionally did not provide answers to the remaining parts of the questions or intentionally left them blank due to a lack of knowledge. Therefore, to avoid such a finding, we might suggest underlining the required parts of the question to draw students’ attention or we might conduct an online test and make all answers required, making it unlikely to have missing answers. In contrast, similar studies have found that the Fresno test is a good tool for assessing competence among EBM novices; however, it was not clear whether they have used the test as an assessment method [24, 25].

Overall, this study supports the evidence that a short EBM module can improve knowledge and skills among undergraduate medical students. This result is consistent with a systemic review that showed that learners’ competence in EBM increased post-intervention across all studies irrespective of the type of educational method [26]. Our curriculum included a mixture of three methods: didactic (lectures), workshop-based and self-directed learning with measurable outcomes. Overall, students were positive about their learning experiences in the workshops. Although workshops are considered a common teaching method in EBM, few published studies have assessed their effectiveness among undergraduate students [27, 28]. In this study, workshops were an effective method to improve EBM technical skills, such as searching the literature, critiquing the validity of findings, and performing statistical calculations, such as sensitivity, specificity, and NNT calculations. The workshop required active participation, and we provided the students with the materials (scenarios, articles and critical appraisal worksheet) prior to the workshop. Additionally, the study was conducted with a small group guided by a facilitator with a facilitator-student ratio of 1 to 12. Our objectives were focused on developing critical appraisal skills with particular attention to results interpretation and statistical concepts. These same objectives were defined in a similar study performed on healthcare professionals that showed that the more active the participation, the more knowledge trainees gained [29].

One might question the sustainability and extent to which courses can actually be responsible for learners’ improved EBM practice after they finish the course. There is no clear evidence on the best timing and duration of EBM teaching. One study recommended the inclusion of EBM courses at least twice during medical studies, with greater intensity shortly before graduation [30]. Other studies recommended a longitudinal EBM course [31] or a course offered in the clinical years [32]. However, the current study cannot answer the question of when EBM courses should be offered. Such a limitation is to be expected with a before-and-after design, which supports weaker inferences than a randomized trial. Future studies with randomization and a sufficiently long follow-up after intervention might provide better insight into this issue. A strength of our study is its development of a new assessment method for EBM competence, i.e., the EBM assignment. However, such tools require further validation, which will be performed in an upcoming study. Thus far, we are satisfied that we succeeded in incorporating EBM as part of the curriculum in clinical years. We have perceived that students are using these skills in other clinical courses that require them to present updated topics, prepare for student-led seminars/tutorials or simply present their research in symposia and conferences.

We believe that there are areas of future improvement and even major changes in our MBBS curriculum provided that the current curriculum has academic accreditation. Until then, we’ll continue cycle of improvement based on EBM module evaluation and feedback from both faculty and students.

## Conclusion

EBM is an important competence for undergraduates. This study supports that a short course in EBM that is incorporated into the undergraduate curriculum, especially in the clinical years, might be effective in improving medical students’ knowledge and skills in EBM. Furthermore, this study adds to the body of evidence that suggests multiple teaching and learning strategies that can improve students’ short-term EBM knowledge and skills. However, rigorous studies are necessary to assess the long-term impact of these interventions and ultimately their effectiveness for clinical decision making.

## Data Availability

All data used in the study are available for interested researchers upon request from the corresponding author after approval from the Institutional Review Board at PNU (contact irb@pnu.edu.sa).

## Abbreviations

ARR: Absolute risk reduction
EBM: Evidence-based medicine
EBP: Evidence-based practice
PNU: Princess Nourah bint Abdulrahman University
PICO: An EBM term for formatting answerable questions (population, intervention, comparison, outcome)
SDL: Self-directed learning
LR: Likelihood ratio
NNT: Number needed to treat)
RRR: Relative risk reduction
